# LDL Receptor-Related Protein-1 (LRP1) Regulates Cholesterol Accumulation in Macrophages

**DOI:** 10.1371/journal.pone.0128903

**Published:** 2015-06-10

**Authors:** Anna P. Lillis, Selen Catania Muratoglu, Dianaly T. Au, Mary Migliorini, Mi-Jeong Lee, Susan K. Fried, Irina Mikhailenko, Dudley K. Strickland

**Affiliations:** 1 Center for Vascular and Inflammatory Diseases, University of Maryland School of Medicine, Baltimore, MD 21201, United States of America; 2 Department of Physiology, University of Maryland School of Medicine, Baltimore, MD 21201, United States of America; 3 Department of Surgery, University of Maryland School of Medicine, Baltimore, MD 21201, United States of America; 4 Department of Medicine, Section of Endocrinology, Diabetes and Nutrition, Boston University School of Medicine, Boston, MA 02118, United States of America; 5 Department of Pathology, Duke University Medical Center, Durham, NC 27710, United States of America; University of Basque Country, SPAIN

## Abstract

Within the circulation, cholesterol is transported by lipoprotein particles and is taken up by cells when these particles associate with cellular receptors. In macrophages, excessive lipoprotein particle uptake leads to foam cell formation, which is an early event in the development of atherosclerosis. Currently, mechanisms responsible for foam cell formation are incompletely understood. To date, several macrophage receptors have been identified that contribute to the uptake of modified forms of lipoproteins leading to foam cell formation, but the contribution of the LDL receptor-related protein 1 (LRP1) to this process is not known. To investigate the role of LRP1 in cholesterol accumulation in macrophages, we generated mice with a selective deletion of LRP1 in macrophages on an LDL receptor (LDLR)-deficient background (macLRP1-/-). After feeding mice a high fat diet for 11 weeks, peritoneal macrophages isolated from *Lrp* ^+/+^ mice contained significantly higher levels of total cholesterol than those from macLRP1-/- mice. Further analysis revealed that this was due to increased levels of cholesterol esters. Interestingly, macLRP1-/- mice displayed elevated plasma cholesterol and triglyceride levels resulting from accumulation of large, triglyceride-rich lipoprotein particles in the circulation. This increase did not result from an increase in hepatic VLDL biosynthesis, but rather results from a defect in catabolism of triglyceride-rich lipoprotein particles in macLRP1-/- mice. These studies reveal an important *in vivo* contribution of macrophage LRP1 to cholesterol homeostasis.

## Introduction

Key events during the development of atherosclerosis include the accumulation of lipoprotein particles in the subendothelial arterial intima, where they are taken up by recruited monocytes/macrophages [1]. This process occurs via interaction of cholesterol ester-rich lipoprotein particles with specific macrophage receptors that target the internalized lipoproteins to lysosomes. Here the lipoprotein particles are processed and the cholesterol esters are hydrolyzed to free cholesterol [2]. In cells, excess free cholesterol is subsequently re-esterified and stored in the cytoplasm as lipid droplets, which under conditions of excessive uptake results in the morphologic appearance known as foam cells [3].

There is considerable interest in identifying macrophage receptors that participate in the internalization of lipoprotein particles leading to cholesterol accumulation *in vivo*. Early studies confirmed that native forms of LDL particles are not taken up by macrophages and therefore do not contribute to excessive cholesterol uptake in these cells [3–5]. It was subsequently discovered that modified forms of LDL are internalized by macrophages leading to unregulated cholesterol uptake [3,4,6]. Further investigation revealed that macrophages express a number of scavenger receptors that recognize oxidized forms of LDL. Cell based studies have identified two members of this receptor family, SR-A (*Msr1*) and CD36 (*Cd36*) that can mediate the cellular internalization of oxidized LDL leading to cholesterol ester accumulation and foam cell formation [7]. Peritoneal macrophages isolated from *Cd36* ^-/-^, *apoE* ^*-/-*^ double knockout mice or *Msr1* ^-/-^, *apoE* ^*-/-*^ double knockout mice on a high fat diet show a marked reduction in free and esterified cholesterol [8] confirming the *in vivo* importance of these receptors in cholesterol accumulation. However, targeted deletion of both SR-A and CD36 on an apoE-deficient background does not abrogate macrophage foam cell formation, revealing that other mechanisms exist in macrophages that contribute to lipid uptake [9]. More recently, the lectin-type oxidized LDL receptor (LOX1) has been identified in endothelial cells as well as macrophages [10] which binds oxidized forms of LDL, and enhances the development of atherogenesis in LDL receptor-deficient mice [11].

Certain modifications of LDL, such as incubation with secreted sphingomyelinase, results in aggregation of the LDL particles which leads to enhanced macrophage uptake and cholesterol loading in these cells [12–14]. Various mechanisms have been suggested for the internalization of aggregated LDL particles by macrophages [15], including receptor-mediated uptake by the LDL receptor-related protein 1 (LRP1) [16–18]. LRP1 is a large endocytic receptor that was originally identified when Herz et al. [19] cloned a large protein containing multiple LDLa repeats and when Ashcom et al. [20] and Moestrup et al. [21] isolated and sequenced the liver receptor responsible for catabolism of α_2_-macroglobulin (α_2_M)-proteinase complexes [22]. In addition to its ability to bind α_2_M-proteinase complexes, early cross-linking studies revealed that LRP1 can also bind apolipoprotein E-containing liposomes [23] suggesting that LRP1 might also function as a receptor for chylomicron and VLDL remnants rich in apoE. This was confirmed in studies revealing that genetic deletion of hepatic LRP1 in LDL receptor-deficient mice resulted in a substantial increase in remnant accumulation in the plasma [24].

LRP1 is abundant in several cells, including macrophages. Prior work has revealed that mice with a selective deletion of LRP1 in macrophages have more extensive atherosclerosis when crossed into an apoE/LDL receptor double knockout mouse [25] or when bone marrow from LRP1-/- mice are transplanted into irradiated LDL receptor-deficient mice [26–28]. Additionally, genetic deletion of LRP1 in macrophages also results in more extensive vascular remodeling upon injury [29]. The mechanisms by which macrophage LRP1 modulates the development of atherosclerosis and the extent of vascular remodeling are not fully understood at this time, but may involve LRP1’s ability to regulate the phagocytosis of apoptotic cells [27,28], its ability to regulate the TGF-β signaling pathway [29], or its ability to modulate macrophage migration by coordinating with the integrin Mac-1, tissue-type plasminogen activator and its serpin inhibitor, PAI-1 [30]. The current studies were undertaken to determine the contribution of macrophage LRP1 to cholesterol homeostasis and foam cell formation by employing mice with tissue-selective deletion of the *Lrp1* gene in macrophages. The results reveal an important contribution of macrophage LRP1 to cholesterol uptake in macrophages.

## Materials and Methods

### Animals

Mice with LRP1 deleted in macrophages were generated on an LDLR-deficient background by crossing LysMCre mice [31] (kindly provided by I. Förster, Munich) with *LDLR* ^-/-^, *LRP1* ^*flox/flox*^ mice [24] (kindly provided by J. Herz, Dallas) as described [32]. Littermate siblings of Cre-, termed LRP1+/+ (*LDLR* ^*-/-*^, *LRP1* ^*flox/flox*^, *Cre* ^*-/-*^) or Cre+, termed macLRP1-/- (*LDLR* ^*-/-*^, *LRP1* ^*flox/flox*^, *Cre* ^*-/+*^) were used in all experiments. Mice were genotyped by polymerase chain reaction (PCR) as described [29]. Mice were weaned at 3 weeks, maintained on a 12-hour light/12-hour dark cycle and fed standard rodent chow (4% wt/wt fat, Harlan Teklad) and water *ad libitum*. For lipoprotein studies, mice were placed on a “Western” diet (21% wt/wt fat, 0.2% wt/wt cholesterol, Harlan Teklad TD-88137) for the indicated times. All animal experiments were approved by the University of Maryland School of Medicine Animal Care and Use Office.

### Macrophage isolation

Peritoneal macrophages were harvested from macLRP1-/- and LRP1+/+ mice four days after intraperitoneal injection of 1 ml thioglycollate broth (Brewer modified; 5% wt/vol; Becton Dickinson) by flushing the peritoneum with 3x5 ml ice cold PBS. Macrophages were washed with ice cold PBS and incubated in DMEM containing 10% fetal calf serum and penicillin/streptomycin in 10 cm cell culture plates at 37°C. Bone marrow derived macrophages were generated as described [32].

### Antibodies and immunoblotting

Anti-LRP1 R2629 has been described [22]. Anti-apoE antibodies were purchased from AbCam. Rabbit polyclonal anti-apoC3 was obtained from Santa Cruz, while anti-Mac-2 IgG was purchased from Cedarlane laboratories. For immunoblotting, cell extracts were prepared as described [33]. 30 μg of protein from each lysate was resolved by SDS-PAGE under non-reducing conditions on 4–12% Tris-glycine SDS gels. Proteins were transferred to nitrocellulose, and membranes were blocked with 3% milk in Tris-buffered saline for 1 hour. Membranes were incubated for 1 hour at room temperature with the indicated antibody at 1 μg/ml in Tris-buffered saline with 3% milk and 0.05% Tween 20. Membranes were washed, incubated with HRP-conjugated goat-anti-rabbit secondary antibody (1:10,000) and then washed and developed with ECL Reagents (Pierce), or by incubation with an appropriate IRDye (LI-COR Biosciences) conjugated secondary antibody. In this case, immunoreactive bands were detected using LI-COR's Odyssey Infrared Imaging System.

### Lipoprotein analysis

Blood samples were drawn from mice following fasting by retro-orbital sinus bleeds. Serum was assayed for total cholesterol (Cholesterol E kit, Wako Diagnostics) and total triglycerides (Sigma) using commercially available spectrophotometric kits. Lipoproteins from pooled serum were separated on a Superose 6 FPLC column run at a flow rate of 0.5 ml/min. 100 μl of serum was loaded on the column and 0.5 ml fractions were collected. The cholesterol and triglyceride content of each fraction was determined as above. Plasma lipoproteins from LRP1+/+ and macLRP1-/- mice fed the aforementioned Western diet for 3 weeks were isolated by sequential floatation ultracentrifugation as described [34]. Briefly, blood from retro-orbital sinus bleeds was collected in EDTA-containing tubes (final concentration 4 mM) and plasma was separated by low speed centrifugation at 4°C. Samples of plasma from two LRP1+/+ and two macLRP1-/- mice were pooled. After the plasma was adjusted to a density of 1.019 g/ml with NaBr, aliquots of 1 ml were transferred to the bottoms of centrifuge tubes. Each was overlaid with 4 ml of a NaBr solution of density 1.019 g/ml. Following 20 h of ultracentrifugation in a SW 55TI rotor (Beckman) at 34,000 rpm and at 16°C, 0.5 ml fractions were collected from the top of the tubes (labeled VLDL fraction). Protein content of these fractions was determined using the BCA assay (Pierce). For SDS-PAGE analysis, 15 μg was separated on a 4–20% Tris-glycine gel under reducing conditions and stained with Coomassie blue. To measure the apoC3 content of the VLDL fraction, acetone precipitation was performed by adding 1 volume (40 μg of protein) to 4 volumes of ice cold acetone and incubating for 2 h at -20°C. All samples were centrifuged, and the pellets were dissolved in SDS reducing buffer and resolved on a 4–20% Tris-glycine gel. Proteins were transferred to nitrocellulose, and membranes were blocked with 3% milk in Tris-buffered saline for 1 h. Membranes were incubated overnight at 4°C with either rabbit polyclonal anti-apoC3 (1 μg/ml in TBS with 3% milk and 0.05% Tween 20) or rabbit polyclonal anti-apoE (1 μg/ml in TBS with 3% milk and 0.05% Tween 20). Immunoreactive bands were detected and analyzed as described above.

For the gavage experiments, 13 week old male mice who had been fed standard chow diet were fasted before olive oil gavage (10 ml/kg; Sigma-Aldrich) [35]. Blood samples were obtained by tail snipping at the indicated times. Serum was analyzed with Serum TG Determination Kit (Sigma-Aldrich) according to the manufacturer’s protocol.

### Binding and uptake of ^125^I-labeled α_2_M* and aggregated LDL by macrophages

Freshly isolated thioglycollate-elicited peritoneal macrophages were plated on 12-well plates. Iodination of trypsin-activated α_2_M (α_2_M*) and uptake experiments were performed as described [36]. Aggregated LDL was prepared by vortexing DiI-labeled LDL in PBS at room temperature as described [18]. Aggregated LDL was incubated with macrophages cultured in 5% lipoprotein-free serum for 24 h at 37°C. Following incubation, cells were washed and the amount of LDL internalized was measured by fluorescence microscopy.

### Cholesterol assays in macrophages

Thioglycollate-elicited peritoneal macrophages (2x10^6^ cells) isolated from macLRP1-/- and LRP1+/+ mice were plated in 100 mm dishes in serum free media for 3 h at 37°C. Cells were scraped off the plate into 4 ml PBS and centrifuged at 1000 rpm for 5 min. Cell pellets were extracted with 300 μl isopropanol with sonication (2 x 10 s, medium setting). The cell extract was centrifuged at 12000 rpm for 5 min and the supernatant was removed and assayed for cholesterol. Total cholesterol and free cholesterol was determined using the Invitrogen Amplex Red Cholesterol Assay in the presence or absence of cholesterol esterase respectively. Samples were diluted 10- to 40-fold before assaying. The cell pellet was taken up in 50 μl 4M guanidinium hydrochloride and the protein concentration determined using the Pierce BCA assay.

### Cholesterol efflux assay

Cholesterol efflux assays were carried out essentially as described [37]. Thioglycollate-elicited peritoneal macrophages were seeded in 24-well plates at a density of 3 x 10^5^ cells/well overnight in Macrophage Growth Medium (MGM; DMEM supplemented with 10% FBS and 10% L-929 conditioned medium). Non-adherent cells were removed, and adherent cells were washed twice with PBS and cultured in MGM. The cells were incubated with 50 μg/mL oxidized LDL (Alfa Aesar) and 1 μCi/mL [1,2-^3^H(N)]-cholesterol (PerkinElmer) in MGM for 24 hours at 37°C. Following incubation, the cells were washed twice with PBS, incubated in low serum growth medium (DMEM supplemented with 1% FBS, penicillin, and streptomycin), and treated with or without 8-Br-cyclic AMP (Sigma) for 16–18 hours. To induce efflux, cells were washed twice with PBS and incubated in DMEM containing 50 μg/mL HDL (Intracel). At the indicated time points, the medium was collected and centrifuged at maximum speed for 10 minutes and cells were harvested by addition of NP-40 lysis buffer. Radioactivity in the medium and cell lysate was quantified by liquid scintillation counting, and percent cholesterol efflux was calculated as follows: (cpm_Media_/(cpm_Media_ + cpm_Cell_)) x 100 after subtracting background efflux (efflux in the absence of HDL).

### In vivo hepatic VLDL-triglyceride production

After a 4 hour fasting period, mice (macLRP1-/-, n = 11 and LRP1+/+, n = 10) were injected with 500 mg/kg body weight Triton WR1339 (Tyloxapol 0.15g/ml; Sigma) in 0.9% NaCl. At the indicated timepoints following injection, blood samples were obtained by tail snipping and the serum was assayed for triglycerides as described above.

### Heparin releasable LPL and apoE from macrophages

Cultured bone marrow-derived or thioglycollate-elicited macrophages were kept in serum free media overnight and then incubated with heparin (50 U/ml) for 30 min at 37°C. Lipase activity was measured as described below. For apoE immunoblot analysis, StrataClean beads (Stratagene) were added to the media. The samples were centrifuged, incubated with reducing SDS sample buffer, and heated at 95°C for 3 min. The samples were then resolved on 4–20% Tris-glycine gels.

### Lipase assays

Fasted LRP1+/+ or macLRP1-/- mice were euthanized after 3 weeks on the Western diet or a chow diet. Livers, epididymal fat pads, gastrocnemius and soleus muscles were isolated, and were incubated with heparin (50 U/ml) on ice for 45 min to elute lipoprotein lipase bound to cell surface heparin-sulfate proteoglycans. Lipase activities were determined as previously described [38].

### Gene expression analysis

Total RNA was extracted from thioglycollate-elicited peritoneal macrophages using Trizol (Invitrogen) reagent as directed by the manufacturer. 1 μg total RNA was used to synthesize cDNA by using the First Strand cDNA Synthesis Kit (SABiosciences). Quantitative real-time PCR was performed on an ABI 7900 HT instrument (Applied Biosystems) by using RT^2^ Real-Time SYBR Green/ROX PCR Master Mix (SABiosciences). Gene expressions were normalized to beta glucuronidase, (*Gusb)*, hypoxanthine guanine phosphoribosyl transferase *(Hprt1)* and heat shock protein 90 alpha *(Hsp90ab1)* housekeeping genes. Data were analyzed based on ΔΔC_t_ fold-change method.

### Statistics

The two-tailed unpaired Student’s *t*-test was used to analyze significance between two groups (LRP1+/+ vs macLRP1-/-). In the instance when the influence of two independent variables (Time and genetic variation) on one dependent variable (TG levels) was analyzed, a two way ANOVA analysis was performed using GraphPad PRISM software. In all statistical analysis, p-values less than 0.05 were considered significant. The Figure Legends indicate which test was performed.

## Results

### Effective tissue-specific Lrp1 gene deletion in Kupffer cells

**Fig 1:**
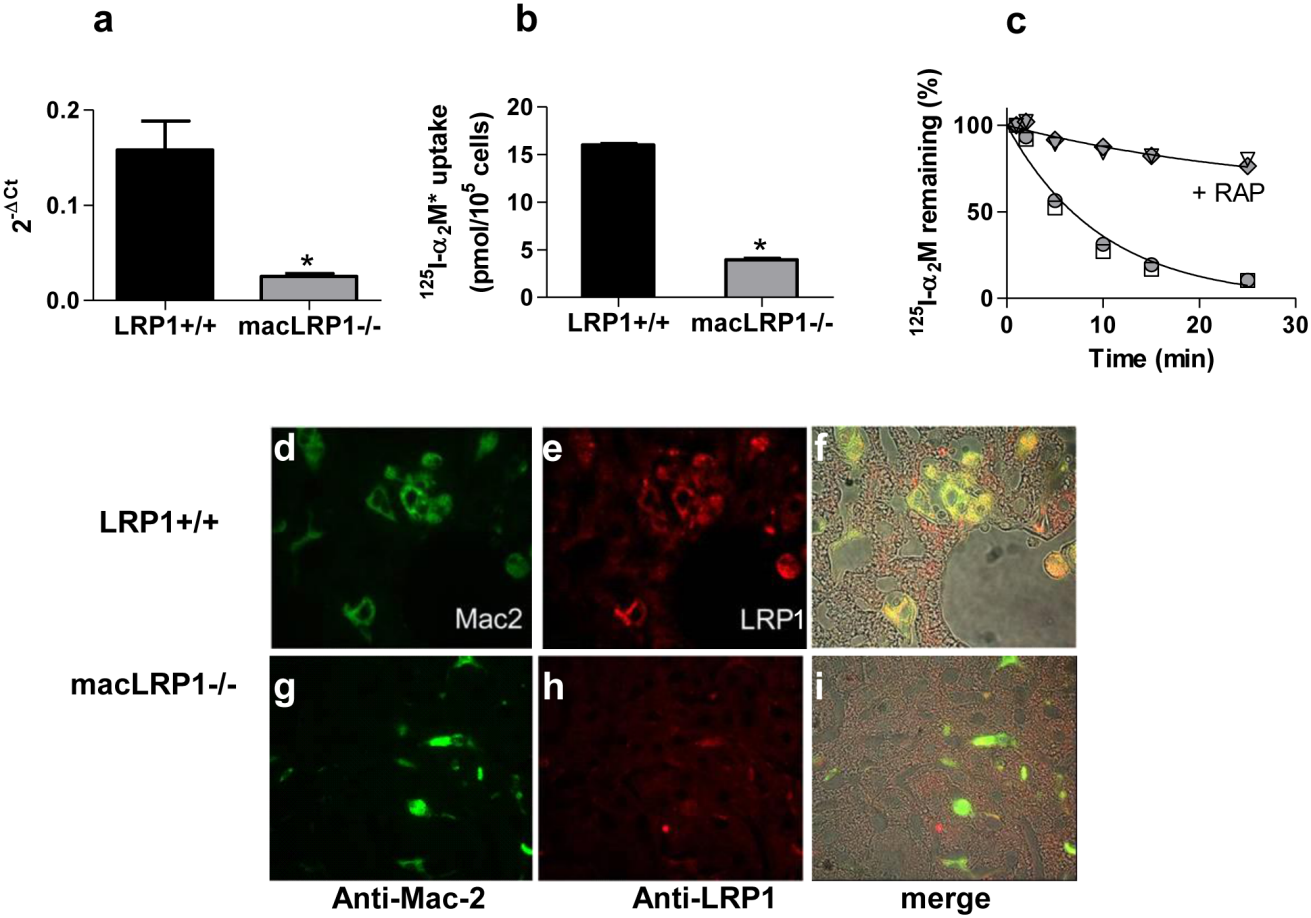
Effective deletion of the *Lrp1* gene in elicited macrophages, bone marrow-derived macrophages, and Kupffer cells. (**a**) Quantitative real-time PCR was performed on bone marrow derived macrophages to determine *Lrp1* levels (**p = 0.005, Student’s t-test comparing LRP1+/+ with macLRP1-/-, n = 3 independent experiments). (**b**) Elicited peritoneal-derived macrophages from LRP1+/+ and macLRP1-/- mice were measured for their ability to internalize activated forms of ^125^I-labeled α_2_-macroglobulin (*p = 0.003, Student’s t-test comparing LRP1+/+ with macLRP1-/-, n = 2 independent experiments). (**c**) macLRP1-/- (open symbols) and LRP1+/+ (closed symbols) mice were injected with ^125^I-α_2_M* alone or with excess RAP. Plasma was sampled over time and counted. Counts were normalized to the amount of radioactivity detected at 1 min following injection. (^125^I-α_2_M*, n = 6; ^125^I-α_2_M* + RAP, n = 3). Error bars denote SEM. (**d-i**) Formalin-fixed paraffin-embedded liver sections from Western-fed LRP1+/+ (**d-f**) and macLRP1-/- mice (**g-i**) were stained with Alexa 488-anti-Mac-2 (**d,g**) and Alexa 546-anti-LRP1 (**e,h**) antibodies. Fluorescent channels merged with phase contrast images of the tissue (**f,i**) show yellow co-staining of LRP1 and Mac-2 in LRP1+/+ livers (**f**). No co-staining was observed in the macLRP1-/- livers (**i**). Livers from three sibling pairs of mice were stained. Representative fields from one pair are shown.

In the current experiments, effective deletion of LRP1 in macrophages was confirmed by quantitative real time PCR (Fig 1a). In addition, we measured the functional activity of LRP1 in elicited peritoneal macrophages by assessing their ability to mediate the internalization of ^125^I-labeled α_2_M*. The results reveal a substantially diminished ability of thioglycollate-elicited peritoneal macrophages from the macLRP1-/- mice to internalize this LRP1 ligand, confirming loss of functional activity in macrophages from macLRP1-/- mice (Fig 1b). These results revealed an effective and reproducible ablation of LRP1 expression in macrophages isolated from macLRP1-/- mice consistent with our previous results [29,32].

Resident liver macrophages, known as Kupffer cells, also express LRP1. To evaluate Kupffer cell LRP1 expression in LRP1+/+ and macLRP1-/- mice, immunohistochemistry was performed on sections of formalin-fixed paraffin-embedded livers from mice fed a Western diet for four weeks. Tissues were co-stained for LRP1 and the Kupffer cell marker, Mac-2, using an Alexa 546-conjugated rabbit polyclonal anti-LRP1 IgG, and an Alexa 488-labeled rat monoclonal anti-Mac 2 antibody. The results (Fig 1d–1i) revealed co-localization of Mac-2 and LRP1 staining in the LRP1+/+ mice, but no co-localization was detected in the macLRP1-/- mice. As anticipated, the results confirm that resident macrophages are depleted of LRP1 in macLRP1-/- mice. To ensure that hepatic LRP1 function was normal in these mice, we examined the clearance of ^125^I-labeled α_2_M* in LRP1+/+ and macLRP1-/- mice. This LRP1 ligand is known to be taken up by hepatocytes [39]. The results confirm normal hepatocyte-mediated clearance of ^125^I-labeled α_2_M* in macLRP1-/- mice that is effectively blocked by the receptor associated protein, a potent LRP1 antagonist (Fig 1c). Together, these data reveal an effective ablation of LRP1 antigen and function in thioglycollate-elicited peritoneal macrophages and Kupffer cells in the macLRP1-/- mice, but normal LRP1 function in hepatocytes.

### Deletion of LRP1 in macrophages reduces accumulation of cholesterol esters in vivo

**Fig 2:**
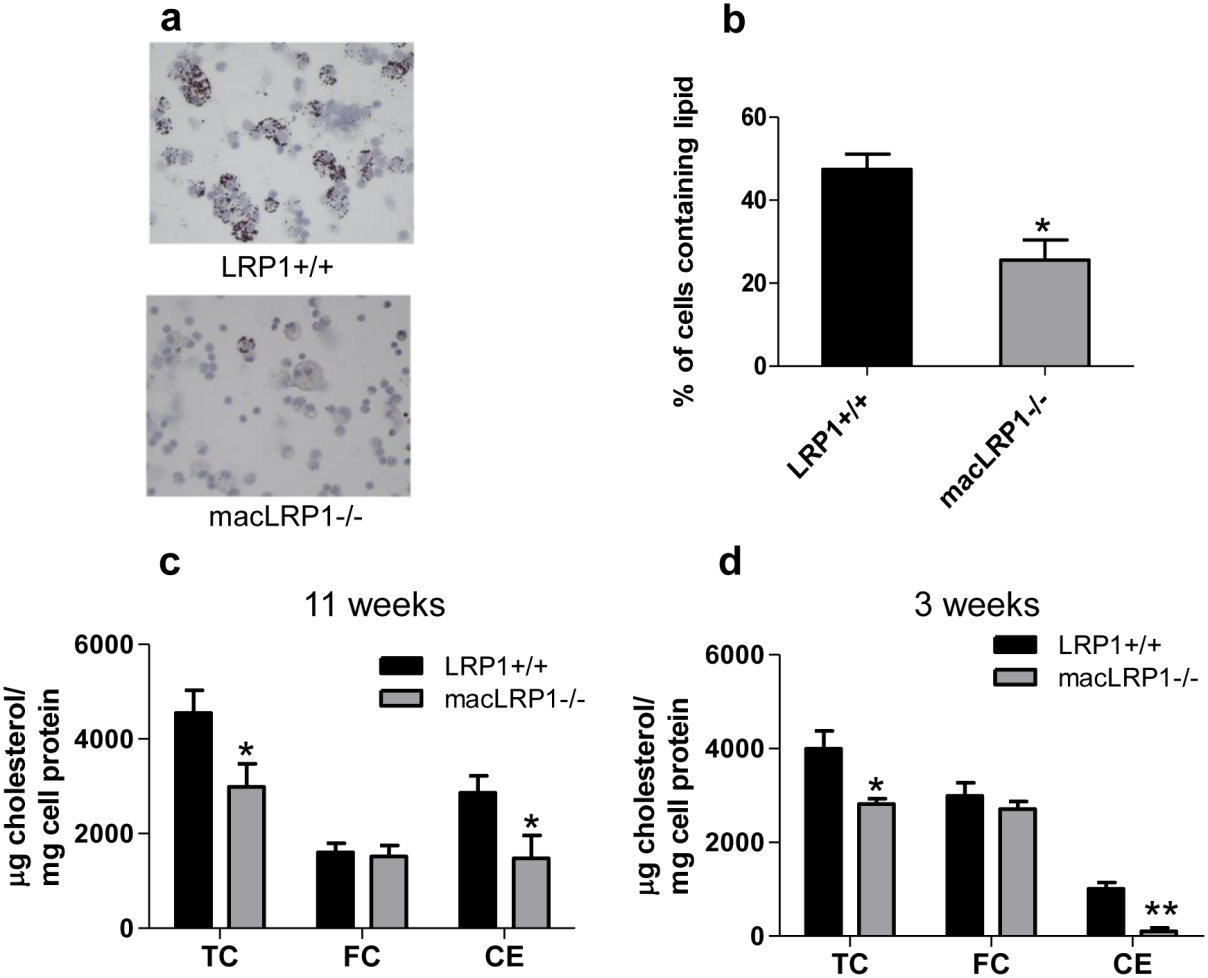
Peritoneal macrophages from macLRP1-/- mice are defective in cholesterol ester accumulation. (**a**) LRP1+/+ (*upper panel*) or macLRP1-/- mice (*lower panel*) were fed a Western diet for 11 weeks. After this period, thioglycollate-elicited peritoneal macrophages were analyzed by Oil red O staining. (**b**). Quantification of the percentage of cells containing Oil red O positive staining. Macrophages from LRP1+/+ (n = 3) and macLRP1-/- (n = 3) were pooled and stained with Oil red O. Four fields for each genotype were counted. (*p = 0.0016, Student’s t-test comparing LRP1+/+ with macLRP1-/-). (**c,d**) Thioglycollate-elicited peritoneal macrophages from LRP1+/+ and macLRP1-/- (n = 3 for both) were isolated from mice following 11 weeks (**c**) or 3 weeks (**d**) on a Western diet, and cholesterol levels were measured. (* p<0.05, **p<0.004, Student’s t-test comparing LRP1+/+ with macLRP1-/-, n = 7). TC, total cholesterol; FC, free cholesterol; CE, cholesterol ester.

To determine if LRP1 influences cholesterol accumulation in macrophages *in vivo*, we harvested macrophages from LRP1+/+ and macLRP1-/- mice fed a Western diet for 11 weeks. Compared with macrophages expressing LRP1, macrophages lacking LRP1 demonstrated less Oil Red O staining (Fig 2a and 2b) and 34% less total cholesterol (Fig 2c). The increased cholesterol content in macrophages isolated from LRP1-expressing mice resulted from higher levels of cholesterol esters in these cells (Fig 2c). Even when mice were fed a Western diet for shorter periods of time (3 weeks), LRP1 expressing macrophages still accumulated increased amounts of total cholesterol, resulting from increased levels of cholesterol esters (Fig 2d).

### LRP1-deficient macrophages are defective in internalizing aggregated LDL

**Fig 3:**
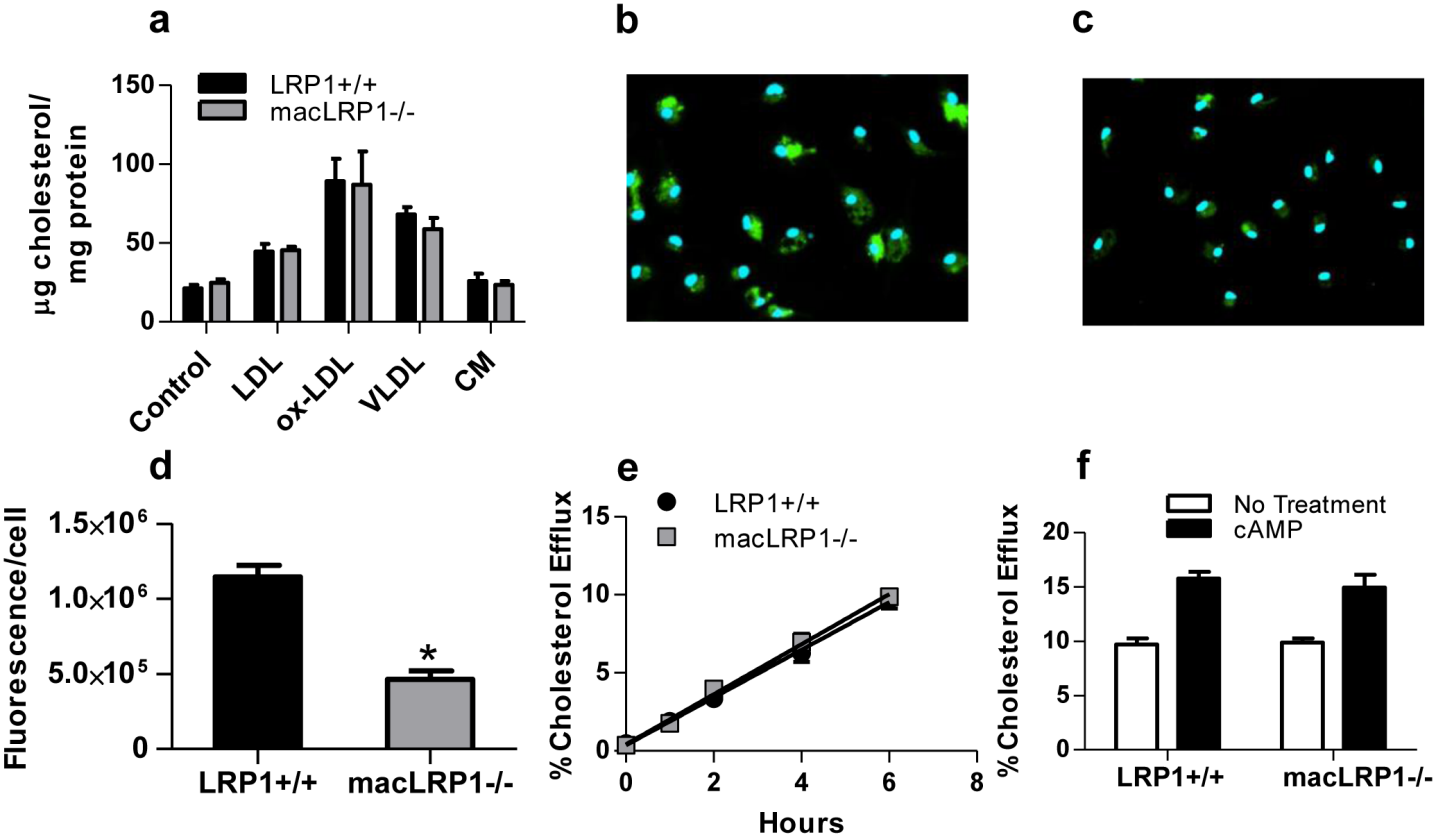
Macrophages from macLRP1-/- mice are defective in internalizing aggregated LDL. **(a)** Thioglycollate-elicited peritoneal macrophages from LRP1+/+ and macLRP1-/- mice were cultured in DMEM supplemented with 5% delipidated fetal calf serum containing 80 μg/ml LDL, oxidized LDL, VLDL, or chlylomicrons for 3 days at 37°C. Lipids were then extracted and total cholesterol was measured and normalized to total cell protein. (**b,c**) Thioglycollate-elicited peritoneal macrophages from LRP1+/+ and macLRP1-/- mice were cultured in DMEM supplemented with 5% delipidated fetal calf serum. DiI-labeled aggregated LDL was then incubated with LRP1+/+ (**b**) or macLRP1-/- macrophages (**c**) for 24 h at 37°C and analyzed for lipoprotein internalization by fluorescence microscopy. (**d**) The extent of internalized fluorescence was quantified using Velocity Software (*p<0.001, Student’s t-test comparing LRP1+/+ with macLRP1-/-) (**e**) Cholesterol efflux of peritoneal macrophages from LRP1+/+ and macLRP1-/- mice (n = 3). (**f**) Cholesterol efflux measured at 6 h in the presence or absence of cAMP (n = 3).

To define potential mechanisms by which macrophage LRP1 mediates the uptake of lipoprotein particles, we examined the ability of peritoneal macrophages to mediate the uptake of cholesterol derived from lipoproteins. Initial experiments were performed in which LRP1-expressing or LRP1-deficient macrophages were cultured in the presence of delipidated serum or delipidated serum containing LDL, oxidized LDL, VLDL, or chylomicrons. After 3 days, the cholesterol content of the macrophages was quantified (Fig 3a). The results reveal no differences between LRP1 expressing and LRP1-deficient macrophages. Several studies have reported the ability of LRP1 to mediate the uptake of aggregated LDL [16–18], which is generated by the action of secreted acid sphingomyelinase [12–14]. We examined the internalization of DiI-labeled aggregated LDL by peritoneal macrophages from LRP1+/+ and macLRP1-/- mice. The results reveal uptake of fluorescently labeled aggregated LDL particles in macrophages from LRP1+/+ mice (Fig 3b). In contrast, LRP1-deficient macrophages are not effective in internalizing aggregated LDL (Fig 3c). Quantitative analysis of the data confirmed significantly more uptake of aggregated LDL particles in macrophages expressing LRP1 (Fig 3d).

**Fig 4:**
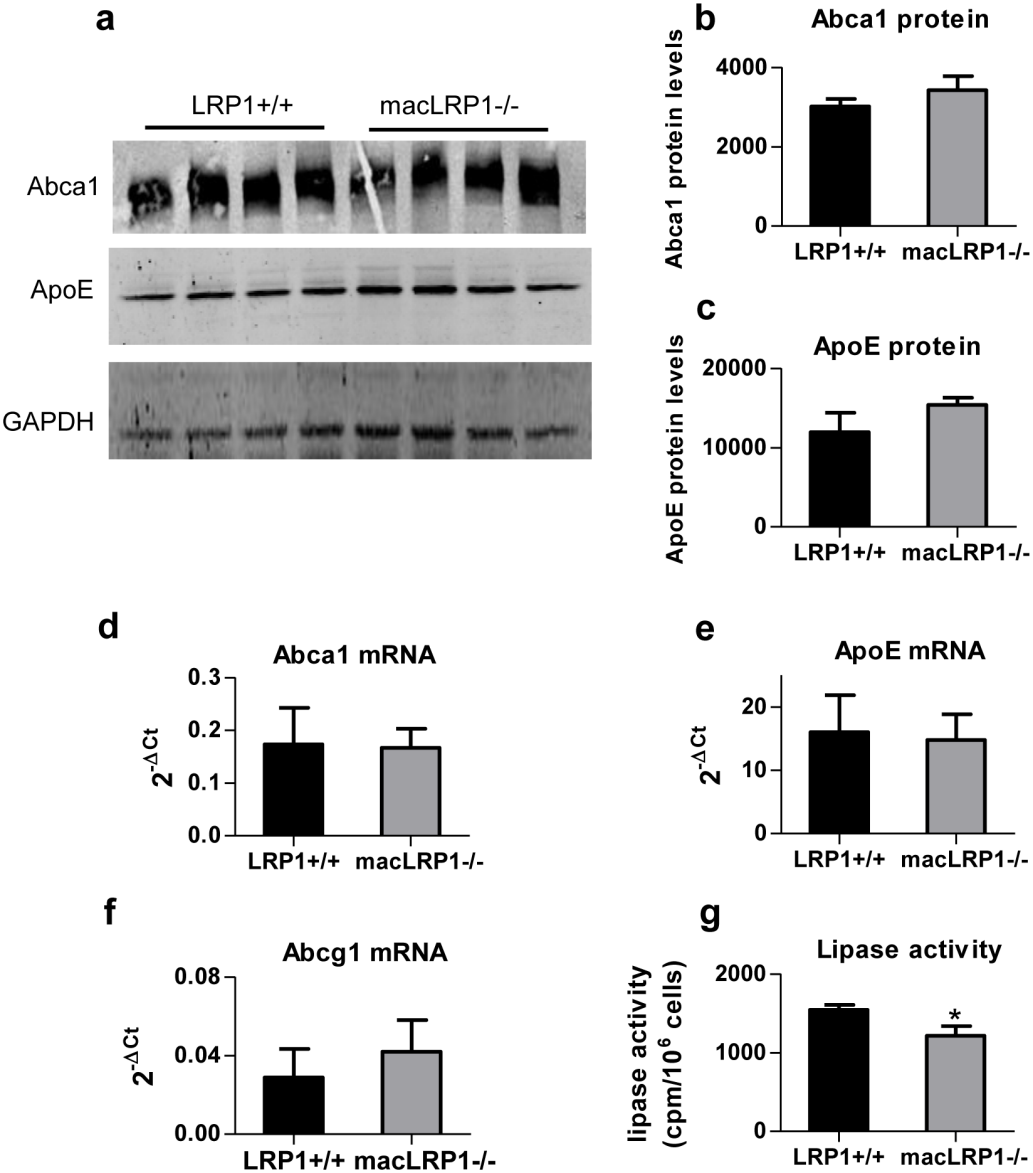
No changes in ABCA1 and ApoE protein and mRNA levels in macLRP1-/- macrophages. (**a**) Cell extracts from thioglycollate-elicited peritoneal macrophages isolated from mice fed a Western diet for 11 weeks were subjected to immunoblot analysis for ABCA1 or ApoE (n = 4). (**b**) ABCA1 protein levels and (**c**) ApoE protein levels normalized to GAPDH were quantified using NIH ImageJ software. (**d,e**) Quantitative RT-PCR was employed to measure levels of *Abca1* (**d**) and *apoE* (**e**) and *Abcg1* (**f**) mRNA (n = 3). (**g**) Lipase activity was measured following heparin elution from LRP1+/+ and macLRP1-/- macrophages. (*p<0.001, n = 4 Student’s t-test comparing LRP1+/+ with macLRP1-/-).

Efflux of cholesterol from macrophages and other cells is mediated by ABCA1 and ABCAG transporters. Prior work in vascular smooth muscle cells revealed that LRP1 modulates levels of ABCA1 by altering the expression of liver X receptors which are key transcriptional regulators of ABCA1 [40]. Thus, we examined cholesterol efflux from peritoneal macrophages isolated from LRP1+/+ and macLRP1-/- mice. The rate of cholesterol efflux was linear up to 6 h, with no differences between macrophages isolated from macLRP1-/- and LRP1+/+ mice (Fig 3e). cAMP is known to induce cholesterol efflux [41], and we measured its effect on cholesterol efflux in macLRP1-/- macrophages. As shown in Fig 3f, cAMP stimulated cholesterol efflux in both LRP1+/+ and macLRP1-/- macrophages, with no differences noted between them. We also measured protein and mRNA levels of *Abca1* and *apoE* in elicited peritoneal macrophages isolated from LRP1-expressing and LRP1-deficient mice that were fed a Western diet for 11 weeks. Consistent with the cholesterol efflux results, we noted no significant differences in ABCA1 and apoE protein (Fig 4a–4c) and mRNA levels (Fig 4d and 4e) in these cells. In addition, we also found that the mRNA levels for the cholesterol transporter ATP-binding cassette transporter G1 (*Abcg1*) were similar in LRP1+/+ and LRP1-deficient macrophages (Fig 4f). Finally, we found no difference in mRNA levels of the ATP-binding cassette transporter 2 (*Abca2*) gene (data not shown).

Lipoprotein lipase (LPL) is abundantly expressed in macrophages, and since macrophage-derived LPL plays a significant role in the regulation of serum triglycerides [42], we measured heparin-releasable lipase activity in culture media from thioglycollate-elicited macrophages in LRP1+/+ and macLRP1-/- mice. The results revealed a 21% decrease in the steady state levels of cell surface lipase activity from LRP1-deficient macrophages when compared to LRP1+/+ macrophages (Fig 4g).

### Elevated remnant lipoprotein levels in the plasma of macLRP1-/- mice

**Fig 5:**
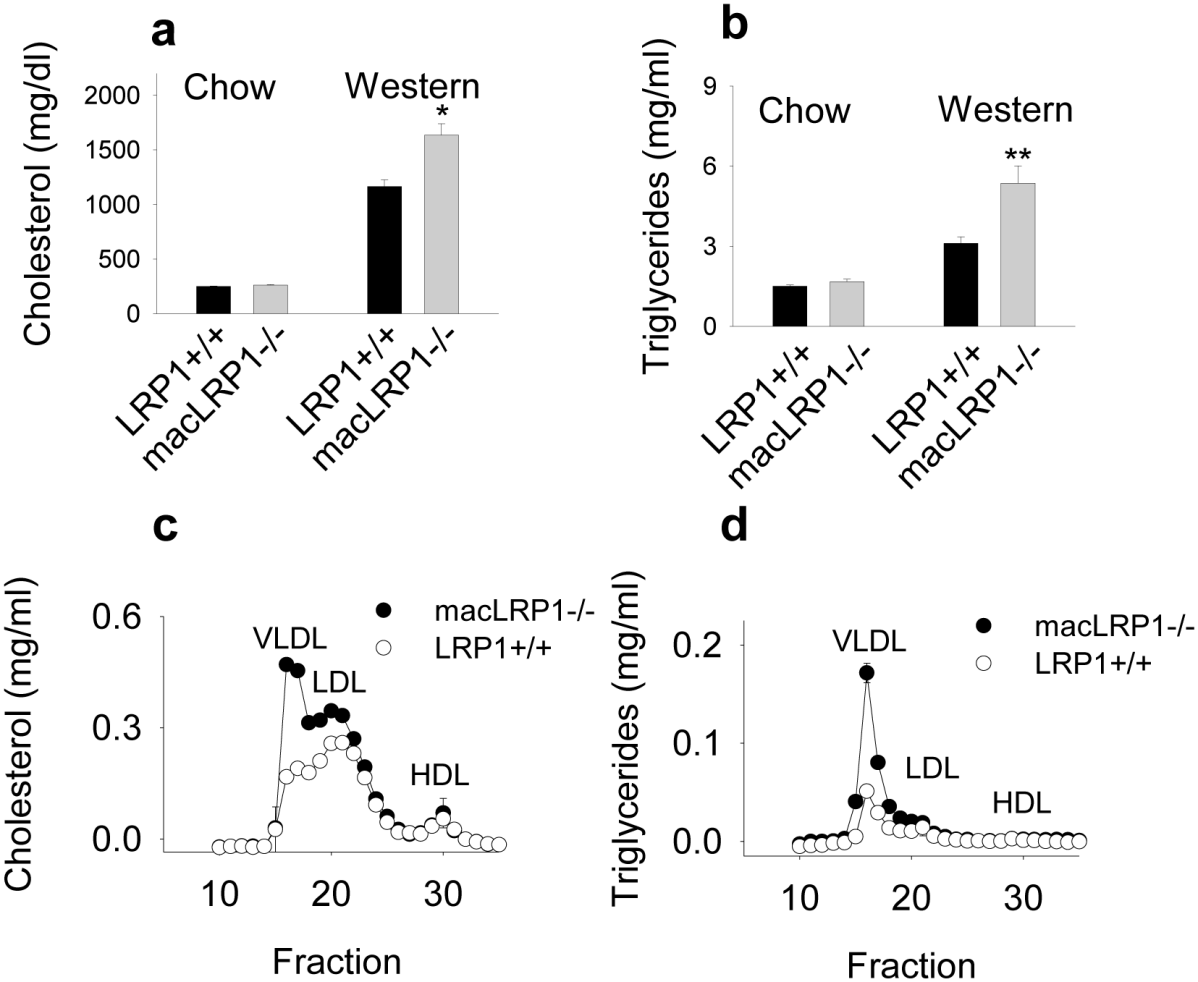
Macrophage LRP1 deficiency leads to accumulation of triglyceride-rich lipoproteins in the plasma of LDLR-deficient mice. Mice were fed either a normal chow (**a**, *left*; **b**, *left*) or Western diet (**a**, *right*; **b**, *right*; **c,d**) for six weeks. Aliquots of serum from fasted mice were assayed for cholesterol (**a**) or triglyceride (**b**) (* p = 0.0004, **p = 0.003, n = 19 per group, Student’s t-test comparing LRP1+/+ with macLRP1-/-). (**c,d**) 100 μl of pooled serum (n = 19/group) from macLRP1-/- and LRP1+/+ mice was fractionated over a Superose 6 FPLC column. Plasma cholesterol (**c**) and triglyceride (**d**) levels were quantified. Three independent experiments were performed, each with a unique cohort of mice. Data from one representative experiment is shown.

To determine if genetic deletion of LRP1 in macrophages alters plasma lipoprotein levels, we fed adult LRP1+/+ and macLRP1-/- mice either a chow or Western diet for 6 weeks and then collected the serum after fasting and measured total serum cholesterol and triglyceride levels. We noted a striking and statistically significant increase in plasma cholesterol (Fig 5a) and plasma triglyceride levels (Fig 5b) in macLRP1-/- mice when compared to LRP1+/+ mice after six weeks on the Western diet.

To identify the lipoprotein fraction(s) that are increased in macLRP1-/- mice, plasma samples from fasted mice were subjected to fast protein liquid chromatography (FPLC) gel filtration chromatography. LRP1+/+ or macLRP1-/- mice fed a chow diet displayed no difference in their lipoprotein profile, either measured by cholesterol content or triglyceride content. Mice fed a Western diet displayed significantly elevated VLDL/LDL levels in the macLRP1-/- mice (Fig 5c and 5d).

**Fig 6:**
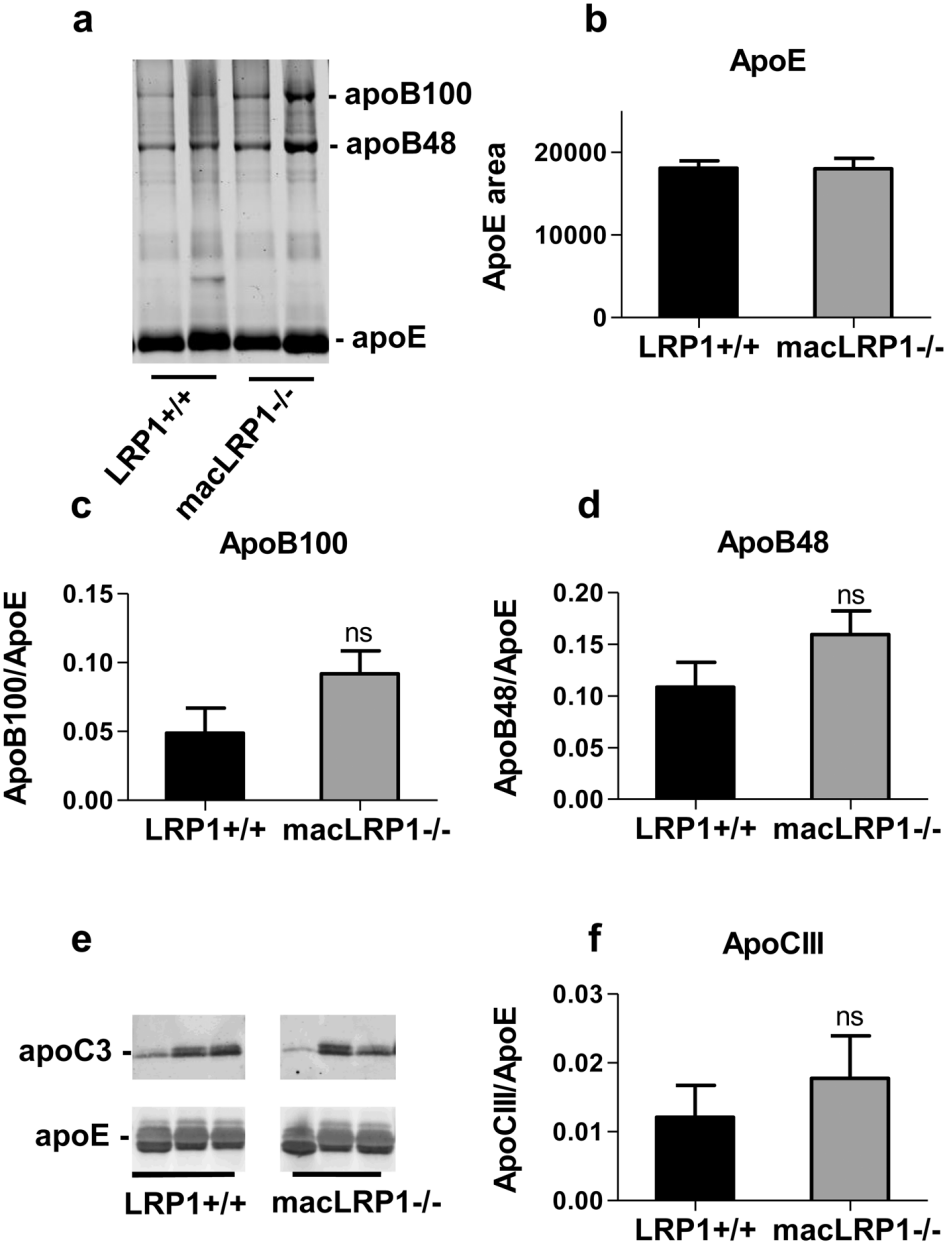
Normal ratios of apolipoproteins in the isolated d<1.019 lipoprotein fraction. The d<1.019 fraction from pooled plasma of LRP1+/+ and macLRP1-/- mice was isolated. (**a**) 15 μg of protein from the d<1.019 fraction was analyzed by SDS-PAGE (n = 2) (**b**) ApoE area was quantified using NIH ImageJ. (**c**) Ratio of apoB100 to apoE and (d) ratio of apoB48 to apoE were determined using NIH ImageJ. (**e**) Immunoblot for apoC3 and apoE content and (**f**) the ratio of apoC3 to apoE in the d<1.019 fraction for LRP1+/+ and macLRP1-/- mice (n = 3). (ns, not significant as determined by Student’s t test comparing LRP1+/+ with macLRP1-/-).

To further characterize the lipoproteins that accumulate in the macLRP1-/- mice, the fraction of lipoproteins with d < 1.019 g/ml was isolated by density gradient centrifugation, and analyzed by SDS-PAGE and immunoblotting. When normalized to protein loading, this analysis revealed no significant changes in the apoE content of the lipoprotein particles (Fig 6a and 6b). In addition, the ratios of apoB100 and apoB48 to apoE were not significantly different in the lipoprotein particles isolated from macLRP1-/- mice when compared to LRP1+/+ mice (Fig 6c and 6d). Studies have shown that excess apoC3 results in hypertriglyceridemia due to delayed catabolism of VLDL and chylomicron remnants [43,44], and thus we examined the levels of apoC3 associated with the d < 1.019 g/ml fraction by immunoblot analysis. These results revealed variable amounts of apoC3 levels associated with these fractions from both LRP1+/+ and macLRP1-/- mice with no significant difference between these groups of mice (Fig 6e and 6f).

### Elevated remnant lipoproteins result from a catabolism defect in macLRP1-/- mice

**Fig 7:**
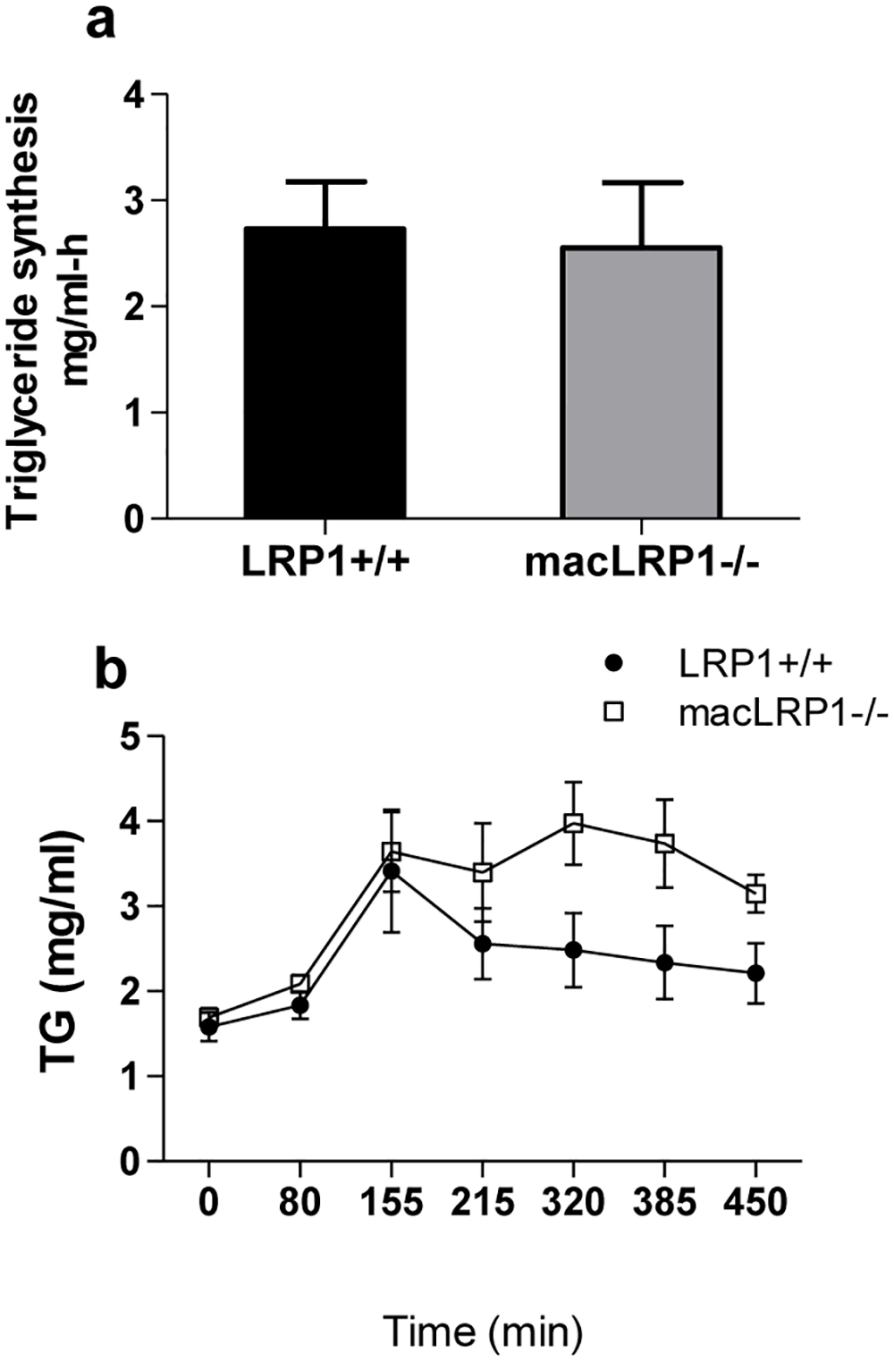
Postprandial serum lipids remain elevated in macLRP1-/- mice. **(a**) Mice (macLRP1-/-, n = 11; LRP1+/+, n = 10) were fed Western diet for 3 weeks, and were then injected with Triton WR-1339 (500 mg/kg body weight), and triglyceride levels in the plasma were determined at 0, 30, 60 and 90 min following injection. The rate of triglyceride synthesis is shown. Error bars show the SEM. (**b**) LRP1+/+ mice (closed symbols, n = 5) or macLRP1-/- mice (open symbols, n = 5) fed a Chow diet were fasted and then received an intragastric olive oil load. Blood samples were collected at the indicated times, and plasma triglyceride levels measured. Error bars show the SEM. (Data were analyzed for statistical significance employing a two way ANOVA which confirmed significant effects for Genotype (p = 0.0012) and Time (p = 0.0001) with no significant Genotype X Time interaction).

Increased accumulation of triglyceride-rich particles in the plasma could result from either increased biosynthesis of VLDL particles from the liver or decreased catabolism. In order to differentiate between the two, we measured the triglyceride biosynthesis rate. This was accomplished by measuring temporal increases in plasma triglycerides under conditions in which triglyceride hydrolysis by lipoprotein lipase is inhibited by injecting the nonionic detergent Triton WR-1339 [45]. The results (Fig 7a) demonstrate that there is no difference in the rate of triglyceride synthesis between LRP1+/+ and macLRP1-/- mice, suggesting that the increase in triglycerides in macLRP1-/- mice is likely due to an alteration in the processing (i.e. lipolysis) and/or the clearance of the particles.

To test this possibility, we measured the plasma triglyceride levels following an intragastric bolus of olive oil administration. The results, shown in Fig 7b, confirm that at 2.5 hours following gavage, the level of triglyceride-rich lipoproteins in the plasma were comparable in both the LRP1+/+ and macLRP1-/- mice, confirming that the VLDL synthesis rates are identical in these mice. Following this period, the levels of triglyceride-rich lipoproteins in LRP1+/+ mice decreased with time as expected. In contrast, however, the plasma triglyceride-rich lipoprotein levels persisted up to 7.5 hours following gavage in macLRP1-/- mice. These results reveal that the catabolism of triglyceride-rich lipoprotein particles is impaired in the macLRP1-/- mice.

### Lipase activity is not altered in muscle, liver and adipose tissue in macLRP1-/- mice

**Fig 8:**
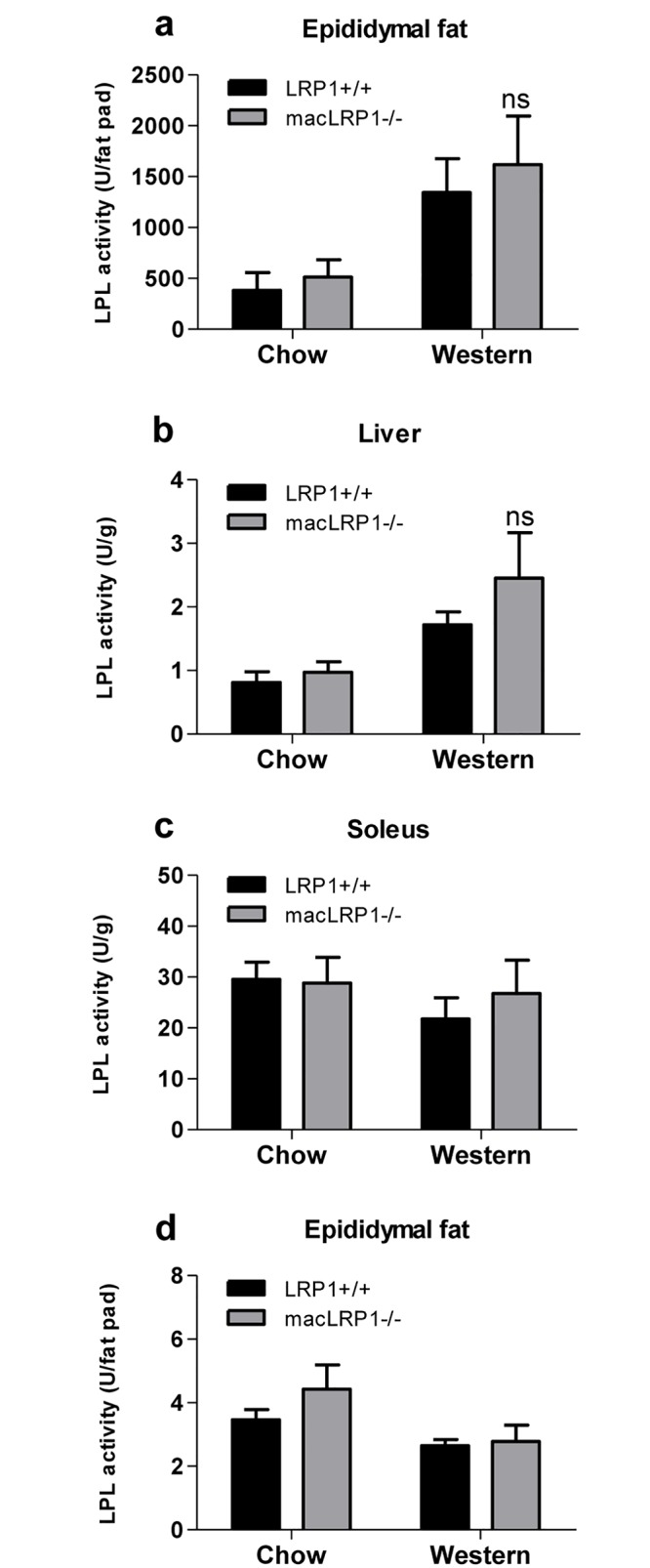
Normal lipase activity in macLRP1-/- mice. Adult mice were fed either Western or normal chow diet for 3 weeks. Following fasting, the epididymal fat (**a**), livers (**b**), soleus muscles (**c**) and gastrocnemius muscles (**d**) were harvested. Lipase was eluted from cell surfaces with heparin, and quantified. (Chow diet: macLRP1-/-, n = 4; LRP1+/+, n = 3; Western diet: macLRP1-/-, n = 6; LRP1+/+, n = 6) (ns, not significant as determined by Student’s t test comparing LRP1+/+ with macLRP1-/-).

Lipolysis of VLDL and chylomicron particles at tissue sites is an important component of the processing of these lipoproteins. This process is catalyzed by lipoprotein lipase (LPL), which not only mediates lipolysis of chylomicrons and VLDL particles, but also binds to LRP1 via its carboxyl-terminal domain and facilitates lipoprotein uptake by this receptor [46–48]. After three weeks on either a chow diet or Western diet, adult mice were fasted and the heparin-eluted lipase activity in various organs was measured. We found no significant difference in lipase activity from any of the organs examined between LRP1+/+ and macLRP1-/- mice (Fig 8a–8d).

## Discussion

The results obtained in the current investigation document an important *in vivo* role for LRP1 in mediating cholesterol uptake in macrophages. This was confirmed by demonstrating that macLRP1-/- mice have a significant decrease in macrophage cholesterol content when fed a Western diet. Further, we noted elevated plasma triglyceride and cholesterol levels in macLRP1-/- mice. Together, these data provide compelling *in vivo* evidence that LRP1 contributes to cholesterol accumulation in macrophages. Interestingly, CD36 and SR-A, two receptors implicated in macrophage cholesterol uptake, also show elevated cholesterol and triglyceride levels when *Cd36*-/- and *Msr1*-/- mice on an apoE null background are fed a Western diet [8]. Thus, it is evident that multiple pathways contribute to cholesterol accumulation in macrophages.

The role of LRP1 in chylomicron remnant catabolism is well established [24], although the involvement of this receptor in this process has been questioned since the contribution of LRP1 to chylomicron remnant uptake is only observed upon LDL receptor deficiency [49]. However, it should be noted that chylomicron remnant particles only accumulate in the circulation when *both* the LDL receptor and LRP1 are deleted in the liver. Thus genetic deletion of either hepatic LRP1 alone [24] or the LDL receptor alone [50] does not lead to accumulation of remnant lipoproteins. These results are consistent with the notion that both receptors contribute to remnant lipoprotein clearance, and when one is absent or defective, the other compensates. Remnant clearance by the liver is a complex process and requires the participation of heparin sulfate proteoglycans, such as syndecan-1 [51]. Further, current models suggest that uptake of remnant lipoproteins by LRP1 requires enrichment of the particle with apoE [52], and studies in LDL receptor as well as apoE-deficient mice suggest that the source of this apoE is the liver [53].

In contrast to the hepatic uptake of lipoproteins, the mechanisms by which macrophages take up lipoprotein particles are not as well understood. Macrophage scavenger receptors have been identified that participate in the uptake and internalization of modified forms of LDL [54–56], and both SR-A (*Msr1*) and CD36 (*Cd36*) have been implicated in this process [7,8]. Despite convincing reports that these two receptors contribute to the development of atherosclerosis and macrophage foam cell formation [57,58], abundant foam cell macrophages were detected in atherosclerotic lesions of mice containing genetic deletions of both receptors [9] revealing that other pathways exist for excessive lipid uptake by macrophages. Our studies suggest that LRP1 also contributes to this process. Several other mechanisms have been proposed that may also contribute to macrophage foam cell formation. For example, Kruth and colleagues have found that LDL can be internalized in macrophages by macropinocytosis [59], and have proposed this as another pathway for macrophage foam cell formation. A response-to-retention hypothesis has also been proposed in which intramural accumulation of lipoproteins and lipoprotein microaggregates represent an important early event in atherosclerosis that leads to modifications of the lipoproteins with important biological consequences including foam cell formation [60].

The findings of elevated triglyceride-rich lipoprotein levels in the plasma of macLRP1-/- mice fed a Western diet are somewhat suprising since our results differ from ealier work on macLRP1-/- mice. For example, Hu et al. [25] generated macLRP1-/- mice on an apoE/LDLR double-deficient background and did not detect any differences in plasma cholesterol and triglyceride levels in their studies when fed a Western diet. It is likely that the absence of apoE expression in their mice accounts for the differences with those of the current study, since LRP1 requires apoE for recognition of VLDL and chylomicron remnant particles [61,62]. Overton et al. [26] also conducted a study in which bone marrow from macLRP1-/- mice was transplanted into lethally irradiated female LDLR-/- recipient mice. Similar to the Hu et al. [25] study, Overton et al. [26] noted no difference in plasma triglyceride and cholesterol levels. The difference between their results and those in the present study likely arise from the fact that *in vivo* replacement of resident macrophages, such as Kupffer cells, upon bone marrow transplantation is a relatively slow process taking several months [63].

The mechanism by which macrophage LRP1 influences the levels of plasma triglyceride-rich lipoproteins is not clear at this time, but several possibilities exist. First, macrophages may play a direct role in the clearance of remnant lipoproteins. In this regard, it is interesting to highlight the studies of Hussain et al. [64,65] who demonstrated that signficant amounts of injected labeled chylomicrons were cleared by perisinusoidal bone marrow macrophages identifying an important contribution of macrophages to chylomicron remnant catabolism. Interestingly, hepatic deletion of LRP1 on an LDL receptor-deficient background does not require feeding a Western diet to prompt accumulation of remnant lipoproteins in the plasma [24], whereas macLRP1-/- mice have to be challenged with an atherogenic diet in order to display elevated remnant lipoproteins. Together, these data would suggest that macrophage LRP1 only plays a critical role in remnant clearance when other tissues are overwhelmed. Secondly, it is possible that the Western diet triggers an LRP1-mediated inflammatory response that signals other tissues to alter their usual role in lipoprotein homeostasis. In this regard, it is interesting to highlight that Kupffer cells are the primary source of hepatic pro-inflammatory and pro-fibrogenic cytokines [66], and accumulation of cholesterol in Kupffer cells results in their activation and conversion to a pro-inflammatory phenotype [67]. LRP1 expressed in Kupffer cells may regulate signaling pathways that in turn could alter lipoprotein catabolism. Additional studies are required to determine if this is the case.

Despite the increased cholesterol content of LRP1-expressing macrophages, studies have revealed that macrophage LRP1 protects the vasculature from the development of atherosclerosis [25–28] and from excessive remodeling upon injury [29]. The fact that excessive cholesterol accumulation in LRP1-expressing macrophages does not exacerbate the development of atherosclerosis likely results from the multifunctional nature of LRP1, which binds over 30 distinct ligands and influences numerous signaling pathways. While the exact mechanisms by which macrophage LRP1 modulates atherosclerosis and vascular remodeling are not fully understood, several possibilities exist. First, in the vasculature, LRP1 regulates the TGF-β signaling pathway by suppressing TGF-β2 gene expression and regulates TGF-β2 protein levels by binding and removing this ligand upon endocytosis [29], which may influence the progression of atherosclerosis. Second, interesting studies have found that LRP1 suppresses macrophage inflammatory response to LPS treatment [68] by a mechanism involving proteolysis of the LRP1 ectodomain followed by γ-secretase-dependent release of the LRP1 intracellular domain (ICD). This domain interacts with interferon regulatory factor 3 (IRF-3) resulting in enhanced nuclear export and degradation of this transcription factor thereby reducing expression of IRF-3 target genes. By suppressing the inflammatory response of macrophages, LRP1 could also influence the development of atherosclerosis. Third, LRP1 also modulates a number of signaling pathways, including the PDGF-signaling pathway [33,69–71], as well as the Wnt-signaling pathway [72], both of which impact the development of atherosclerosis. Together, all of these studies highlight the potential of LRP1 to modulate a variety of signaling pathways with the outcome highly dependent upon the initiating stimulus and cellular context.

In summary, the current investigation has confirmed an important *in vivo* role for macrophages and macrophage LRP1 in modulating cholesterol metabolism. The data further reveal that LRP1 is one of the major receptors that contribute to lipoprotein uptake in macrophages leading to foam cell formation. It is well known that the development of atherosclerosis is a complex process involving multiple genes, and defining the potential of LRP1 to regulate these processes may assist in identifying molecules which could have significant therapeutic potential.
