# Correction: LDL Receptor-Related Protein-1 (LRP1) Regulates Cholesterol Accumulation in Macrophages

**DOI:** 10.1371/journal.pone.0147457

**Published:** 2016-01-21

**Authors:** Anna P. Lillis, Selen Catania Muratoglu, Dianaly T. Au, Mary Migliorini, Mi-Jeong Lee, Susan K. Fried, Irina Mikhailenko, Dudley K. Strickland

The authors issue the following correction in order to cite and discuss previously published in vitro studies on the role of LRP1 that are relevant to this article.

The sentence in the Abstract should read “To date, several macrophage receptors have been identified that contribute to the uptake of modified forms of lipoproteins leading to foam cell formation, but the *in vivo* contribution of the LDL receptor-related protein 1 (LRP1) to this process is not known.”

In addition, the following paragraphs should be added to the Discussion:

In the current study it is not clear what form of lipoprotein is recognized by macrophages in vivo. The potential of LRP1 to mediate the uptake of lipoprotein particles was first suggested in cell-based studies demonstrating that LRP1 mediates the uptake of cholesterol esters derived from apolipoprotein E enriched β-VLDL lipoprotein particles [2]. This early study established two important principals regarding the role of LRP1 in mediating lipoprotein catabolism. First, the study demonstrated a requirement for enrichment of the β-VLDL particles with apolipoprotein E in order to be recognized by LRP1 which led to the sequestration model for LRP1 mediated hepatic uptake of lipoproteins [3]. The in vivo role of LRP1 in chylomicron remnant metabolism was firmly established in 1998 when Rohlmann et al. [4] used a genetic approach to reveal LRP1's role as a chylomicron receptor. A second observation from early studies was the finding that unlike the LDL receptor, LRP1 levels are not reduced when cells are incubated with excess hydroxycholesterol [2]. These results suggested that LRP1-mediated uptake of lipoproteins could lead to foam cell formation, and indeed this was demonstrated to be the case when human monocyte-derived macrophages or vascular smooth muscle cells were incubated with aggregated LDL[5,6].

However, it is highly unlikely that aggregated LDL represents the physiological ligand for LRP1 in the macLRP1-/- / LDLR-/- mice fed a Western diet, since we observed accumulation of triglyceride-rich VLDL particles in the plasma of these mice. This result suggests that some form of VLDL particle is the physiological ligand for macrophage LRP1. Indeed, studies have suggested that LRP1-deficient macrophages are defective in mediating the internalization of VLDL particles [7], although we were unable to reproduce this observation in the current study using thioglycollate-elicited peritoneal macrophages. Very likely, the in vivo uptake of lipoproteins by LRP1 in macrophages is complex, and difficult to reproduce in cell culture experiments.
